# *Lrp5* p.Val667Met Variant Compromises Bone Mineral Density and Matrix Properties in Osteoporosis

**DOI:** 10.1002/jbm4.10741

**Published:** 2023-03-28

**Authors:** Stéphanie Fabre, Morgane Bourmaud, Guillaume Mabilleau, Ruben Goulet, Aude Couturier, Alexandre Dentel, Serge Picaud, Thomas Funck‐Brentano, Corinne Collet, Martine Cohen‐Solal

**Affiliations:** ^1^ INSERM U1132 Bioscar Université de Paris Cité Paris France; ^2^ Department of Rheumatology APHP, Lariboisière Hospital Paris France; ^3^ Université Angers, Nantes Université, Oniris, Inserm U1229 RMeS Angers France; ^4^ Sorbonne Université, INSERM, CNRS, Institut de la Vision Paris France; ^5^ Ophthalmology Department Université de Paris Cité, AP‐HP, Lariboisière Hospital Paris France; ^6^ Molecular Genetic Department Robert Debré Hospital Paris France

**Keywords:** bone density, collagene, fracture, Lrp5, Wnt

## Abstract

Early‐onset osteoporosis (EOOP) has been associated with several genes, including *LRP5*, coding for a coreceptor in the Wnt pathway. Variants in *LRP5* were also described in osteoporosis pseudoglioma syndrome, combining severe osteoporosis and eye abnormalities. Genomewide‐association studies (GWAS) showed that *LRP5* p.Val667Met (V667M) variant is associated with low bone mineral density (BMD) and increased fractures. However, despite association with a bone phenotype in humans and knockout mice, the impact of the variant in bone and eye remains to be investigated. Here, we aimed to evaluate the bone and ocular impact of the V667M variant. We recruited 11 patients carrying the V667M variant or other loss‐of‐function variants of *LRP5* and generated an *Lrp5* ^V667M^ mutated mice. Patients had low lumbar and hip BMD *Z*‐score and altered bone microarchitecture evaluated by HR‐pQCT compared with an age‐matched reference population. Murine primary osteoblasts from *Lrp5* ^V667M^ mice showed lower differentiation capacity, alkaline phosphatase activity, and mineralization capacity in vitro. Ex vivo, mRNA expression of *Osx*, *Col1*, and *osteocalcin* was lower in *Lrp5* ^V667M^ bones than controls (all *p* < 0.01). *Lrp5* ^V667M^ 3‐month‐old mice, compared with control (CTL) mice, had decreased BMD at the femur (*p* < 0.01) and lumbar spine (*p* < 0.01) with normal microarchitecture and bone biomarkers. However, *Lrp5* ^V667M^ mice revealed a trend toward a lower femoral and vertebral stiffness (*p* = 0.14) and had a lower hydroxyproline/proline ratio compared with CTL, (*p* = 0.01), showing altered composition and quality of the bone matrix. Finally, higher tortuosity of retinal vessels was found in the *Lrp5* ^V667M^ mice and unspecific vascular tortuosity in two patients only. In conclusion, *Lrp5* ^V667M^ variant is associated with low BMD and impaired bone matrix quality. Retinal vascularization abnormalities were observed in mice. © 2023 The Authors. *JBMR Plus* published by Wiley Periodicals LLC on behalf of American Society for Bone and Mineral Research.

## Graphical abstract

Lrp5^667M^ mutant mice show low bone mineral density, reduced osteoblast function and hydroxylation of collagen matrix. Matrix failure explained bone fragility in early onset osteoporosis.

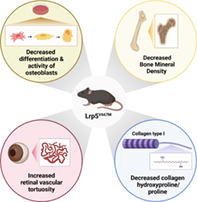

## Introduction

The prevalence of osteoporosis raises with age, postmenopausal status,^(^ ^1^ ^)^ and in the presence of certain pathologies. But age, sex, and medical history explain only 20% to 40% of bone mineral density (BMD) variations,^(^ ^2^ ^)^ whereas heritability has been estimated responsible for 60% to 80% of these variations.^(^ ^3^, ^4^ ^)^ The impact of genetic component is even higher in primary osteoporosis of men and women younger than 50 years, called early‐onset osteoporosis (EOOP). Genomewide‐association studies (GWAS) have identified a large number of genes as associated with low bone mass in the general population,^(^ ^5^ ^)^ some also responsible for monogenic bone disorders.^(^ ^6^ ^)^ The genes involved in the Wnt signaling pathway are particularly represented^(^ ^7^ ^)^ and, more specifically, the *LRP5* (low‐density lipoprotein receptor‐related protein 5) gene. The encoded *LRP5* protein acts as a coreceptor for Wnt ligands to activate the canonical Wnt pathway, known to regulate bone formation.^(^ ^8^ ^)^ Loss‐of‐function mutations of *LRP5* are responsible for osteoporosis pseudoglioma syndrome (OPPG, OMIM 259770),^(^ ^9^ ^)^ a rare autosomal recessive disorder characterized by severe osteoporosis in childhood associated with congenital blindness, whereas gain‐of‐function mutations are responsible for high‐bone‐mass disorders (HBM, OMIM 601884).^(^ ^10^ ^)^ Animal models based on *Lrp5* total and conditional knockouts (KO) have consistently shown decreased BMD,^(^ ^11^, ^12^, ^13^, ^14^, ^15^, ^16^, ^17^ ^)^ and several *LRP5* rare heterozygous variants or homozygous low‐frequency variants have been repeatedly detected in patients with EOOP.^(^ ^18^, ^19^, ^20^ ^)^ Hence, the p.Val667Met (V667M) low‐frequency variant, resulting from the missense single nucleotide mutation c.1999G > A in exon 9 of *LRP5*, has been reported as a genetic risk factor for fractures and low BMD in the general population and is overrepresented in patients with EOOP.^(^ ^4^, ^18^, ^20^, ^21^ ^)^ Obligate carriers of *LRP5* variant had reduced bone mass when compared with age‐ and sex‐matched controls.^(^ ^9^, ^22^ ^)^ This homozygous variant was linked to reduced activation of the canonical Wnt signaling pathway in site‐directed mutagenesis experiments.^(^ ^18^ ^)^ Indeed, heterozygous harboring of this variant is associated with low BMD and EOOP but with a large heterogeneity in the severity of osteoporosis.^(^ ^18^, ^20^, ^21^, ^23^ ^)^ Its contribution in bone fragility remains unclear as a causal relationship between the presence of the variant and clinical outcomes.

*LRP5* is also essential for the development of the retinal vasculature through a variant of Norrin/β‐catenin pathway.^(^ ^24^, ^25^ ^)^ Mutations of *LRP5* are responsible for loss of vision in the OPPG syndrome and other familial exudative vitreoretinopathy (FEVR).^(^ ^26^, ^27^ ^)^ Previous studies reported ocular features in young adults with heterozygous *LRP5* variants.^(^ ^28^, ^29^ ^)^ However, whether osteoporotic patients have vitreoretinopathy or any vessel abnormalities remains unclear. Here, we aimed to characterize the bone and eye phenotype in patients with EOOP and in a mice model harboring the p.Val667Met variant.

## Materials and Methods

### Participants

We analyzed patients younger than 55 years who were referred to the clinic for osteoporosis and/or history of fracture. We excluded patients taking medications that interfered with bone metabolism. Extensive radiological and biochemical investigations allowed excluding secondary causes of osteoporosis. Tests included serum levels of total calcium, phosphorus, 25OH vitamin D and parathyroid hormone, electrophoresis of proteins, C‐reactive protein, thyroid‐stimulating hormone, and serum creatinine. Patients were <55 years old at diagnosis and had a BMD *Z*‐score <−2 standard deviation (SD) at the spine or total hip^(^ ^21^, ^30^ ^)^ associated with osteoporotic fractures. For each patient, the following data were collected: age at diagnosis, number and site of fractures, family history of fracture, and bone marker levels before any treatment. None had signs of skeletal dysplasia or known eye abnormality. In this study, we included patients with *LRP5* suspected pathogenic variants, who were offered a bone and eye evaluation. Clinical and genetic evaluation was part of the assessment of rare bone diseases. Written informed consent was obtained from all participants.

### DNA sequencing and multiplex ligation‐dependent probe (MLPA) technologies

DNA was analyzed as described elsewhere.^(^ ^18^ ^)^ Briefly, DNA samples were screened by targeted gene sequencing panel for variants in the following genes: *LRP5*, Plastin 3 (*PLS3*), Collagen type I α1 (*COL1A1*), Collagen type Ι α2 (*COL1A2*), Wnt family member 1 (*WNT1*), LDL receptor related protein 6 (*LRP6*) and dickkopf Wnt signaling pathway inhibitor 1 (*DKK1*), *WNT3A*, TNF superfamily member 11 (*TNFSF11*), TNF receptor superfamily member 11a (*TNFRSF11A*), TNF superfamily member 11b (*TNFRSF11B*), and vitamin D receptor (*VDR*). All variants were confirmed by Sanger technology. Variant pathogenicity was evaluated by using the prediction software Polyphen (http://genetics.bwh.harvard.edu/pph2), Mutationtaster (http://www.mutationtaster.org), and SIFT (http://sift.bii.a-star.edu.sg). Only the genes in the panels were investigated without other copy number variation (CNV) analysis.

### Biochemical markers of bone

Blood samples were collected to assess bone‐remodeling biomarkers. We used the following methods to measure biomarkers: C‐terminal telopeptide of type I collagen (CTX), N‐terminal propeptide of type I collagen (P1NP), Osteocalcin (Cobas e601 analyzer, Roche Diagnostics, Indianapolis, IN, USA), bone alkaline phosphatase (Ysis analyzer, IDS, Boldon, UK), and TRAP5B levels by ELISA kit (IDS).

### Microarchitecture analysis by HR‐pQCT

Microarchitectural parameters were assessed at the right distal radius and tibia by HR‐pQCT (XtremeCT, Scanco Medical AG, Brüttisellen, Switzerland), as previously described. The resolution (voxel size) was fixed at 80 μm. Quality control was performed by daily scans of the manufacturer's phantom. The volume of interest (VOI) was automatically separated into trabecular and cortical regions using a threshold‐based algorithm (Scanco software version, V 6.1) to provide all parameters of interest.^(^ ^31^ ^)^

### Mice generation

The mutation in humans is caused by a c.1999G > A transition in the codon GTG, coding for valine, resulting in an exchange for methionine (ATG) in position 667 of the protein. We introduced the equivalent mutation in mice within exon 9 in position 1996, resulting in the p.Val666Met mutation. We used a targeting vector, containing two homology regions of 5.1 and 2.8 kb of *Lrp5*, generated by PCR using C57Bl/6N BAC DNA as template, which was linearized and electroporated into embryonic stem cells where it underwent homologous recombination. We used an FRT‐flanked neomycin resistance cassette, inserted in an unsuspicious region in intron 9 that was later excised by Flp recombination, upon breeding with germline Flp‐expressing mice (“Flp‐deletors”). The FLp‐mice used were gray flp‐deleter mice in C57/Bl6 background (C57BL/6‐Tg(CAG‐Flpe)2Arte). Mice obtained with the homozygous targeted mutation were thereafter called *Lrp5* ^V667M^ mice and were analyzed.

All animal experiments were performed according to procedures approved by the local animal ethics committee and were approved by the French Ministry of Higher Education and Research (APAFIS#15223–2018052211589557).

### Culture of primary osteoblasts

Primary osteoblasts were isolated from calvariae of 2‐ to 4‐day‐old control (CTL) and *Lrp5* ^V667M^ pups. Briefly, calvariae were digested for 10 × 2 minutes with 0.2% type IV collagenase (Sigma‐Aldrich, St. Louis, MO, USA) in phosphate‐buffered saline (PBS) with 4 mM EDTA, and then for 45 minutes with 0.2% collagenase in PBS for osteoblast release. The cells were expanded for 5 to 6 days in minimum essential medium‐alpha containing 10% fetal calf serum and plated at a density of 50,000 cells per well in 12‐well culture plates. Two separate experiments were performed in triplicate for each group. The culture medium was supplemented with 50 μm ascorbic acid and 10 mM beta‐glycerophosphate, which was replaced every 2 to 3 days. RNA was extracted for osteoblast gene expression. Determination of alkaline phosphatase activity was performed through alkaline phosphatase staining after 14 days of culture in osteogenic medium, using SigmaFast BCIP/NBT tablets (Sigma). Mineralization was assessed by alizarin red staining after 21 days of culture in osteogenic medium. Mean intensity was measured with the ImageJ software in arbitrary units and normalized to the mean value obtained for CTL.

### Reverse transcription and real‐time quantitative PCR

mRNA was extracted using the Isolate II RNA Mini Kit (Bioline, London, UK), in accordance with the manufacturer's instructions. For each sample, 1 μg of total mRNA was reverse transcribed using the High Capacity cDNA Reverse Transcription Kit (Applied Biosystems, Carlsbad, CA, USA) following manufacturer's instructions. Samples were subjected to quantitative PCR with SensiFAST SYBR (Bioline) on a Light Cycler 480 thermocycler (Roche). Primers will be available upon request. Results were analyzed with the ΔΔCt method, and reference gene was *Sdha* (Succinate Dehydrogenase Complex Flavoprotein Subunit A).

### Serum biomarkers in mice

CTX and P1NP were measured in mice serum with ELISA method with the RatLaps (CTX‐I) EIA (IDS) and Rat/Mouse PINP EIA (IDS) kits, following the manufacturer's instructions.

### Histology and histomorphometry

The right femur from each animal was collected after microCT measurement, dehydrated in xylene, and then embedded without demineralization in methylmethacrylate. Five micrometer‐thick coronal sections were cut parallel to the long axis of the femur, using an SM2500S microtome (Leica, Wetzlar, Germany). Sections of 5 μm were stained with toluidine blue to quantify the osteoblastic, osteoid, and osteoclastic surfaces (Ob.S/BS, OS/BS, and Oc.S/BS, respectively). Two 11‐μm‐thick unstained sections were taken for measurement of the dynamic parameters under UV light. The matrix apposition rate (MAR) was measured using the Microvision image analyzer (Evry, France) by a semiautomatic method using tetracycline and calcein double‐labeled bone surfaces. The mineralizing surfaces (MS/BS) were measured in the same areas using the objective eyepiece Leitz integrate plate II, and the bone formation rate was calculated using the two measured parameters. All the histomorphometric parameters were recorded in compliance with the recommendation of the American Society for Bone and Mineral Research Histomorphometry Nomenclature Committee.^(^ ^32^ ^)^

### Dual‐energy X‐ray absorptiometry

Dual‐energy X‐ray absorptiometry (DXA) analysis of all animals was performed to determine the bone mineral density at the total body (excluding head), lumbar spine, and femurs. The measurement was performed on the animals under anesthesia with a Faxitron device (Hologic, Marlborough, MA, USA).

### Bone structure analysis by microcomputed tomography

Right femurs and L_4_ vertebrae were collected for bone microarchitecture analysis after fixation in 70% ethanol, without decalcification and after inclusion in methylmethacrylate for L_4_ vertebrae. They were analyzed with high‐resolution microCT using a Skyscan 1272 microCT (Bruker, Kontich, Belgium). For femurs, measurements were made on the distal metaphysis for the trabecular bone and on the diaphysis for the cortical bone. Images were obtained using the following acquisition parameters: voltage 75 kV, intensity 125 μA, pixel size 6 μm, exposition time 1350 ms, and filter aluminum 0.25 mm. Images for L_4_ vertebrae were obtained for measurements on the trabecular bone with the following acquisition parameters: voltage 90 kV, intensity 111 μA, pixel size 6 μm, exposition time 1200 ms, filter aluminum 0.5 mm. NRecon, DataViewer, CTAn, and CTVox softwares were used for 3‐dimensional (3D) image reconstruction, analyzed, and expressed according to international guidelines.^(^ ^33^ ^)^ The following morphometric parameters were computed: bone volume/tissue volume (BV/TV, %), trabecular thickness (Tb.Th, μm), trabecular number (Tb.N, 1/μm), trabecular separation (Tb.Sp, μm), cortical thickness (Ct.Th, μm), and cortical area (Ct.Ar, μm^2^).

### Assessment of bone strength

Left femurs and L_2_ vertebrae were collected after euthanization and immediately frozen at −20°C, in a compress soaked in saline solution. Samples were thawed in physiological saline solution 24 hours before mechanical tests at 4°C. The 3‐point bending test was used for femurs, with the Instron 5942 device (Instron, Elancourt, France). The load of the actuator was applied in the center of the shaft at a speed of 2 mm/min until fracture. The span of the lower support was 10 mm. The load–displacement curve was recorded with the Bluehill 3 software (Instron), providing the stiffness, which was defined as the slope of the linear elastic deformation, the yield load at the boundary between the elastic deformation and plastic deformation, and the ultimate load, which is the maximum sustained force at the point of fracture.^(^ ^34^ ^)^ Ultimate displacement and post‐yield displacement were also computerized, as well as work to fracture. The yield load was calculated with the 0.2% offset method. Elastic modulus was estimated through the beam theory. Vertebral strength was assessed by a compression test in the cranio‐caudal axis of L_2_ vertebrae, with a loading speed of 1 mm/min until fracture.

### Analysis of bone composition

Quantitative Backscattered Electron Imaging (qBEI) experiments were performed on right midshaft femurs embedded in methylmethacrylate. The blocks were carbon‐coated and observed with a scanning electron microscope (EVO LS10, Carl Zeiss Ltd, Nanterre, France) equipped with a five‐quadrant semiconductor backscattered electron detector. Method details are provided in Appendix S1. The following physicochemical parameters were determined from spectra: mineralization (mineral‐to‐matrix ratio, mineral crystallinity, carbonate content), collagen proline hydroxylation, nanoporosity, relative proteoglycan (PG), and relative pyridinoline content.

### Evaluation of human retina

All patients underwent a detailed ocular exam including best‐corrected visual acuity using a Snellen chart, slit‐lamp biomicroscopy, measurement of intraocular pressure (IOP) and autorefractometry (TonoRef II; Nidek, Gamagori, Japan), and retinal imaging. All eyes underwent ultrawide‐field color fundus photographs images obtained using Optos (Dunfermline, UK) and optical coherence tomography (OCT)‐angiography (OCTA). Capillary density (CD) was quantified in OCTA image and was converted into binary images using ImageJ (NIH, Bethesda, MD, USA). CD was calculated as the ratio of the area occupied by vessels divided by the total image area. Details can be found in the Appendix S1.

### Evaluation of mouse retina

Eyes were collected immediately after death, fixed in 4% paraformaldehyde overnight, washed, and then stored in PBS at 4°C.

#### Retinal vascularization on flat‐mounted retinas

Eyes were dissected to obtain flat‐mounted retinas, immersed in permeabilization and saturation buffer during 1 hour at room temperature, and then incubated overnight with 1/400 anti‐COLIV goat antibody solution (Bio‐Rad, Hercules, CA, USA; 134001) and revealed using Donkey anti‐goat 488 antibodies (Thermo Fisher Scientific, Waltham, MA, USA; A11055). They were mounted with spacers and a Permafluor mounting medium. Images were obtained using confocal microscopy (Inverted Confocal Olympus, Tokyo, Japan) at ×20 magnification (focal aperture 621 × 621 μm). Three regions of interest (ROI) were picked in peripheral retina. A 3‐dimensional modeling software (Imaris 9.9, Abingdon‐on‐Thames, Oxfordshire, UK) was used to model a total volume of COLIV‐labeled retinal vessels. Vascular tortuosities were marked manually, allowing for volume quantification. Because atrophies were difficult to model, atrophic segments were counted in each ROI, regardless of their volume.

#### Retinal inflammation or glial activity on retinal slices

Eyes were dissected to obtain eyecups that were cryoprotected and frozen. Serial transversal slices (12 μm thick) were performed with cryostat (CL3050 S, Leica), then immersed in permeabilization and saturation buffer during 1 hour at room temperature, incubated overnight with 1/400 Iba1 goat (Invitrogen, Carlsbad, CA, USA; AB5076) and 1/500 GFAP rabbit (Dako, Glostrup, Denmark; Z0334) antibody solutions and revealed using Donkey anti‐goat 488 and anti‐rabbit 594 antibodies (Thermo Fisher Scientific; A11055). Images were obtained using confocal microscopy (Inverted Confocal Olympus) at ×40 magnification.

### Statistical analysis

Data are expressed as median with interquartile range. Mann–Whitney test was used, except when stated otherwise (2‐factor ANOVA variance analysis). The GraphPad (La Jolla, CA USA) software was used for analysis. A difference was considered as significant when *p* < 0.05.

## Results

### EOOP patients with *LRP5* variants have low cortical and trabecular BMD and altered bone microarchitecture

**Fig. 1:**
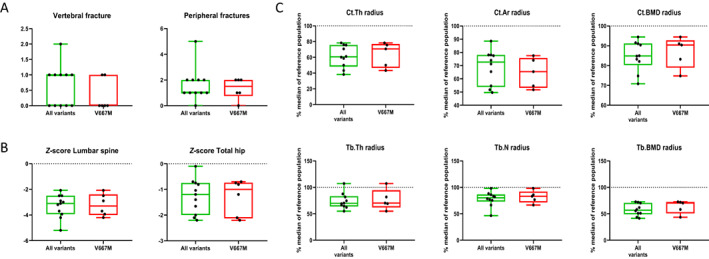
Characteristics of early‐onset osteoporosis (EOOP) patients carrying *LRP5* variants. (*A*) Number of vertebral and peripheral fractures. (*B*) Lumbar spine and hip bone mineral density (BMD) *Z*‐score. (*C*) Bone microarchitectural parameters obtained by HR‐pQCT at the radius and expressed as a percentage of the median value of a reference population, for all EOOP patients with a *LRP5* variant and for patients with the V667M variant only. Ct.Ar = cortical area; Ct.BMD = cortical BMD; Ct.Th = cortical thickness; Tb.BMD = trabecular BMD; Tb.N = trabecular number; Tb.Th = trabecular thickness.

We included 11 patients who met the criteria for EOOP. The patients were referred to the bone clinic because of unusual major nontraumatic fracture and/or low BMD discovered before the age of 55 years. Genetic evaluation showed that they carried *LRP5* variants considered as pathogenic and loss‐of‐function. They were 6 patients with the V667M variant (5 heterozygous and 1 homozygous carriers) and 5 patients with other pathogenic variants (Supplemental Table S1). We compared the phenotype of our patients with reference data. First fracture occurred mainly during the third or fourth decades and could be either vertebral or peripheral (Fig. 1*A*). BMD *Z*‐scores were low, predominantly at the lumbar spine site (−3.1 [−3.9 to −2.5] for all variants; −3.3 [−4 to −2.4] for V667M variant) (Fig. 1*B*). HR‐pQCT analysis revealed an altered cortical and trabecular microarchitecture regardless of the *LRP5* variant involved (Fig. 1*C*). Radius cortical thickness and area were low compared with a sex‐ and age‐matched reference population (Ct.Th 60.7% [48.3–75.6] and 70.7% [46.7–76.9]; Ct.Ar 72.6% [53.9–78.1], and 65.4% [53.2–75.8]) as well as trabecular thickness (69.8% [63.9–83.3] and 70.3% [62.1–94.6]) and number (80.3% [73.2–86.6] and 83.2% [71.7–92.1]). Measurements at the distal tibia followed the same pattern. Trabecular and cortical vBMD values were lower than the median of reference (cortical BMD 84.9% [80.3–91.2] and 90.4% [79.0–92.8]; trabecular BMD 56.8% [49.1–70.7] and 70.3% [50.8–72.3]).

Carriers with all variants and V667M variant had no vitamin D deficiency (31.3 ng/mL [24.3–36.3] and 24.5 ng/mL [23.6–29.1], respectively). Serum bone biomarkers were within the reference range: BAP (12.4 μg/L [12.0–14.5] and 12.6 μg/L [10.4–23.0]), P1NP (48.9 ng/mL [39.6–53.2] and 45.7 ng/mL [39.8–83.5]), CTX (339.0 pg/mL [255.0–416.5] and 338.0 pg/mL [255.0–410.0]), TRAP5b (2.6 U/L [2.15–4.0] and 2.8 U/L [2.2–3.8]).

### *Lrp5* ^V667M^ osteoblasts show lower differentiation capacity

**Fig. 2:**
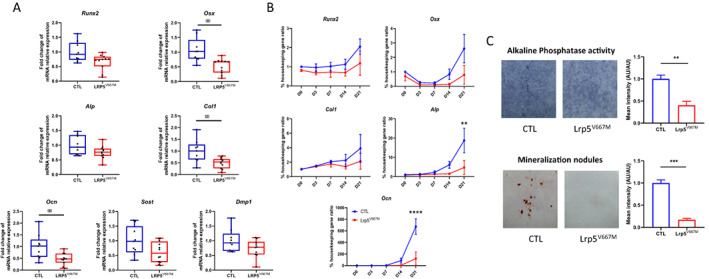
*Lrp5* ^V667M^ primary osteoblasts show lower differentiation capacity. (*A*) mRNA relative expression of *Runx2*, *Osterix* (*Osx*), *Alkaline phosphatase* (*Alp*), *Collagen type 1* (*Col1*), *Osteocalcin* (*Ocn*), *Sclerostin* (*Sost*), and *Dentin matrix acidic phosphoprotein 1* (*Dmp1*) in the tibia of control (CTL) and *Lrp5* ^V667M^ mice. Relative mRNA levels were normalized to the level of Succinate Dehydrogenase Complex Flavoprotein Subunit A (*Sdha*). (*B*) Time course from day (D) 0 to 21 of mRNA relative expression of *Runx2*, *Osx*, *Alp*, *Col1*, and *Ocn* in cultures of calvarial cells extracted from CTL and *Lrp5* ^V667M^ pups. (*C*) Alkaline phosphatase staining at day 14 of culture and red alizarin staining at day 21 of culture with quantification of staining intensity, normalized to the mean intensity of CTL samples. AU = arbitrary unit.

To investigate the impact of the V667M *LRP5* variant, mice carrying the mutation were generated (Supplemental Fig. S1). *Lrp5* ^V667M^ mice (homozygous for the murine equivalent of the V667M variant) were fertile, macroscopically normal, with no difference in size or weight compared with CTL mice. Analysis of long bones without bone marrow showed lower mRNA expression of *Osx*, *Col1*, and *osteocalcin* in *Lrp5* ^V667M^ bones than in CTL ones (all *p* < 0.01) (Fig. 2*A*), suggesting lower differentiation capacity in osteoblasts of *Lrp5* ^V667M^ mice. Murine primary osteoblasts issued from *Lrp5* ^V667M^ mice calvariae also showed decreased differentiation capacity in culture compared with osteoblasts from CTL mice, as illustrated by reduced mRNA expression of alkaline phosphatase (*p* = 0.006 for interaction time × genotype) and osteocalcin (*p* < 0.0001 for interaction time × genotype) (Fig. 2*B*), confirming the effect on osteoblastic differentiation is cell‐autonomous. Alkaline phosphatase activity and mineralization capacity were also reduced in vitro (Fig. 2*C*).

### BMD is lower in *Lrp5* ^V667M^ mice, with similar bone microarchitecture and remodeling

**Fig. 3:**
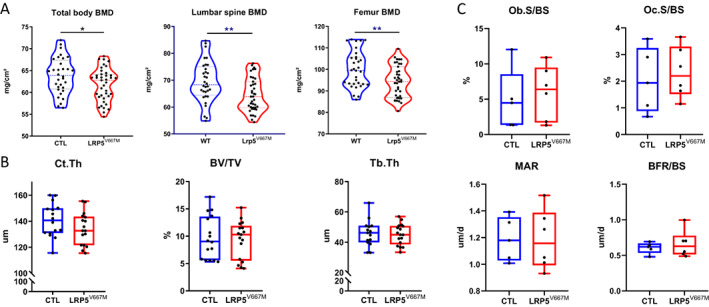
BMD is lower in *Lrp5* ^V667M^ mice, with similar bone microarchitecture and remodeling. (*A*) Bone mineral density (BMD) of 3‐month‐old control (CTL) and *Lrp5* ^V667M^ mice at the total body, lumbar spine, and femur. (*B*) Bone microarchitectural parameters measured with microscanner (Ct.Th = cortical thickness; BV/TV = bone volume/total volume; Tb.Th = trabecular thickness). (*C*) Histomorphometrical static and dynamic bone formation and resorption parameters in CTL and *Lrp5* ^V667M^ mice (BFR/BS = bone formation rate/bone surface; MAR = mineral apposition rate; Ob.S/BS = osteoblastic surface/bone surface; Oc.S/BS = osteoclastic surface/bone surface).

We then analyzed the BMD and microarchitecture in 3‐month‐old mutant and control mice. *Lrp5* ^V667M^ mice, compared with CTL mice, had indeed lower BMD at all the measuring sites (Fig. 3*A*): total body (62.8 mg/cm^2^ [59.0–64.1] versus 64.9 mg/cm^2^ [60.8–67.4], *p* < 0.05), femur (94.3 mg/cm^2^ [86.9–101.2] versus 99.5 mg/cm^2^ [93.2–105.6], *p* < 0.01), and lumbar spine (63.9 mg/cm^2^ [59.7–69.5] versus 68.3 mg/cm^2^ [64.4–75.06], *p* < 0.01). However, microarchitecture parameters at the femur and vertebrae were similar in *Lrp5* ^V667M^ and CTL mice (Fig. 3*B*). At the femur, Ct.Th (132.7 μm [121.6–143.6] versus 140.6 μm [130.9–150.0], *p* = 0.17); BV/TV (10.3% [5.5–11.9] versus 9.0% [5.6–13.6], *p* = 0.9); Tb.Th (45.2 ± 6.9 μm versus 45.9 ± 8.5 μm, *p* = 0.79) were not different, as well as the indices at L_4_ lumbar vertebrae (BV/TV [16.6 ± 2.44% versus 17.02 ± 1.93%, *p* = 0.76]); Tb.Th (45.0 μm [38.7–50.8] versus 46.2 μm [40.0–50.9], *p* = 0.8). In addition, serum bone biomarkers were comparable in the 2 groups (P1NP 57.2 ± 11.8 ng/mL versus 61.5 ± 16.2 ng/mL, *p* = 0.55; CTX 15.8 ± 5.2 ng/mL versus 17.3 ± 3.7 ng/mL, *p* = 0.55) (Supplemental Fig. S2). Histomorphometric analysis revealed no difference in parameters for bone formation (Ob.S/BS 6.39% [1.70–9.49] versus 4.48% [1.37–8.54], *p* = 0.66; OS/BS 18.19% [10.45–30.11] versus 16.59% [7.99–23.47], *p* = 0.79; MAR 1.16 μm/d [0.99–1.39] versus 1.18 μm/d [1.03–1.35], *p* > 0.99; BFR/BS 0.63 μm/d [0.51–0.78] versus 0.62 μm/d [0.53–0.67], *p* = 0.66) or either for bone resorption (Oc.S/BS 2.20% [1.52–3.30] versus 1.94% [0.88–3.25], *p* = 0.66) (Fig. 3*D* and Supplemental Fig. S2).

### Matrix properties are impaired in *Lrp5* ^V667M^ mice but not bone strength

**Fig. 4:**
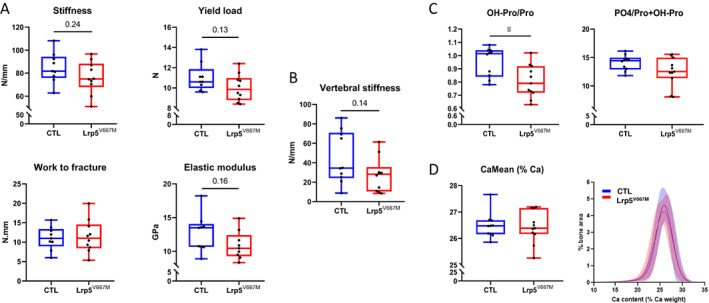
Matrix composition is impaired in *Lrp5* ^V667M^ mice. (*A*) Bone biomechanical properties of femur and (*B*) L_2_ vertebrae of control (CTL) and *Lrp5* ^V667M^ mice. (*C*) Hydroxyproline/proline ratio (OH‐Pro/Pro) and mineral‐to‐matrix ratio (phosphate‐to‐proline+hydroxyproline content, PO4/Pro+OH‐Pro) measured by raman microspectroscopy at sites of bone formation. (*D*) Mean calcium content and distribution of calcium content in bone matrix measured by quantitative backscattered electron imaging (qBEI).

To investigate whether the variant could contribute to bone fragility, we measured the bone biomechanical properties (Fig. 4*A*, *B*). *Lrp5* ^V667M^ mice tended to have a lower femoral and vertebral stiffness (75.0 N/mm [68.0–88.2] versus 81.8 N/mm [75.9–94.2], *p* = 0.24, and 28.2 N/mm [10.6–35.5] versus 34.5 N/mm [24.3–70.8], *p* = 0.14, respectively). For femurs, Young's elastic modulus was estimated and appeared lower than for CTL mice (10.4 GPa [9.3–12.4] versus 13.5 Gpa [10.7–14.0], *p* = 0.16), meaning the altered stiffness does not result from the bones' geometry but rather from modified intrinsic bone biomechanical properties. Work to fracture was similar in the two groups (11.0 N/mm [8.5–14.5] versus 11.0 N/mm [8.9–13.3], *p* = 0.9) but the yield load seemed again lower for *Lrp5* ^V667M^ femurs (9.9 N [8.8–11.0] versus 10.6 N [10.0–11.9], *p* = 0.13), which therefore could undergo plastic nonreversible deformation for lower applied forces and accumulate more microdamage through time, increasing the risk of fracture.

The reduced stiffness suggested altered properties of the extracellular matrix. Indeed, the hydroxyproline/proline ratio, measured in newly formed bone, was lower in *Lrp5* ^V667M^ femurs compared with CTL ones (0.79 [0.72–0.92] versus 1.02 [0.84–1.04], *p* = 0.01). It is possible that the mineral‐to‐matrix ratio was also impacted in *Lrp5* ^V667M^ mice. Indeed, when estimated through the phosphate‐to‐proline+hydroxyproline ratio, it trended to be lower than in CTL mice (12.53 [11.37–14.97] versus 14.45 [12.90–14.98], *p* = 0.2) (Fig. 4*C*) and the pattern was reproduced when other ways to estimate the organic matrix content were used (content in amide 1, amide 3, or amino acid lateral chains of collagen). The calcium content of bones, estimated by qBEI over the full cortical cross section, appeared comparable between *Lrp5* ^V667M^ and CTL mice and distribution of bone areas with high and low calcium content was similar in *Lrp5* ^V667M^ and CTL mice (Fig. 4*D*), suggesting that alterations of bone extracellular matrix observed at sites of bone formation were compensated during maturation of the bone matrix. There was also a trend to higher nanoporosity in *Lrp5* ^V667M^ mice. However, the altered hydroxylation of collagen type 1 chains was not related to a significantly lower expression of prolyl 3‐hydroxylase 1 (*P3H1*) in bone (mRNA expression/Sdha 20.7% [14.0–53.8] in *Lrp5* ^V667M^ mice versus 25.9% [16.1–59.3] in CTL mice, *p* = 0.5) or prolyl 4‐hydroxylase (Supplemental Fig. S3). None of the other physico‐chemical parameters were altered between the two groups of animals.

### Abnormal retinal vascular tortuosity was observed in *Lrp5* ^V667M^ mice

Nine of the 11 *Lrp5* mutated patients underwent the eye examination. Slit‐lamp biomicroscopy examination and measurement of intraocular pressure were normal. The morphology of capillaries in the 3 × 3 mm OCTA images as well as the macular capillary density in the superficial capillary plexus (SCP) and in the deep capillary complex (DCC) were not different from a population of reference (Supplemental Fig. S4). For two patients, one with the heterozygous V667M variant and one with another *LRP5* variant, abnormal retinal vascular tortuosity was present on ultrawide‐field fundus photographs (Supplemental Fig. S4). This increased tortuosity involved first‐order arteries of both eyes in the two patients. Peripheral micro‐hemorrhages were observed in two other patients, one in each group also. However, these anomalies were unspecific. Ultrawide‐field fluorescein angiography did not show significant abnormalities and no peripheral avascularity was observed.

**Fig. 5:**
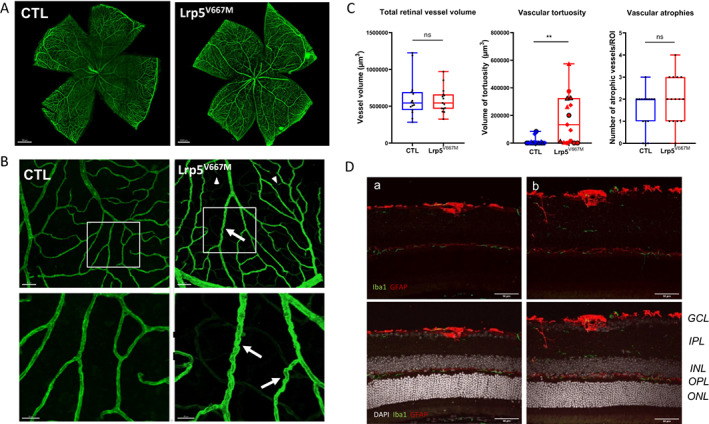
Abnormal retinal vessels in *Lrp5* ^V667M^ mice. (*A*) Whole‐mounted retina of control (CTL) and *Lrp5* ^V667M^ mice stained for COLIV. (*B*) Confocal images of third‐order vessels showing vascular tortuosity (arrow) or atrophic vessels (arrowhead), mostly present in peripheral retina in *Lrp5* ^V667M^ mice. (*C*) Quantification of total volume of retinal vessels, volume of tortuous vessels per region of interest (ROI), and number of atrophic vessels per ROI, estimated from COLIV‐marked retinal vessels in CTL and *Lrp5* ^V667M^ mice. (*D*) Confocal images of 12‐μm retinal slices in CTL (a) and *Lrp5* ^V667M^ (b) mice showing *ionized calcium‐binding adapter molecule 1* (Iba1) and *Glial Fibrillary Acidic Protein* (GFAP) staining. No major difference is evidenced in microglial or astrocytic location or expression measured at the ganglion cell layer (*GCL*), inner plexiform layer (*IPL*), inner nuclear layer (*INL*), outer plexiform layer (*OPL*), or outer nuclear layer (*ONL*).

In mice retinas, total retinal vascular volume was similar in the two groups (Fig. 5*A*, *B*). Vascular atrophy was present in both *Lrp5* ^V667M^ and CTL mice without any significant difference, but the volume of vessels concerned by vascular tortuosity was markedly higher in *Lrp5* ^V667M^ mice (17,3218 μm^3^ for *Lrp5* ^V667M^ versus 12,099 μm^3^ for CTL, *p* = 0.0012) (Fig. 5*B*, *C*), vascular tortuosity being mostly peripheral. Analysis of transversal sections of retinas did not evidence clear modifications of the vascular network in the deeper layers and no strong difference was observed in microglia or astrocyte activation explored through Iba1 or GFAP staining in the ganglion cell layer, suggesting the absence of difference in inflammation or gliosis processes (Fig. 5*D*).

## Discussion

*LRP5* is associated with variations in BMD and risk of fracture. Although heterozygous harboring of V667M variant has been repeatedly described in early‐onset osteoporosis^(^ ^4^, ^18^, ^20^, ^21^ ^)^ and this variant has been suggested as pathogenic, the contribution of *LRP5* variants in the osteoporotic phenotype remains unclear. In addition, the mechanisms of bone fragility are not completely understood. Here, we generated a murine model carrying the *LRP5* p.V667M variant, the most frequently reported as associated with low BMD, and showed that this variant is indeed responsible for a reduced BMD. Mechanisms involved include impairment of osteoblastic differentiation and bone fragility can be explained by modifications of the extracellular matrix.

Here, patients with EOOP carrying *LRP5* variants had low lumbar spine BMD, similarly to previously reported data.^(^ ^18^, ^20^, ^21^, ^35^ ^)^ BMD, measured by DXA and HR‐pQCT, revealed low BMD compared with the general population at the trabecular but also at the cortical compartments, with alterations in bone microarchitecture, as reported in young osteoporotic patients without mention of *LRP5* variants.^(^ ^20^, ^36^, ^37^, ^38^, ^39^, ^40^, ^41^ ^)^ Bone remodeling markers are usually normal or low,^(^ ^18^, ^20^, ^35^, ^36^, ^37^, ^38^, ^39^, ^42^ ^)^ as observed in our patients.

Our rodent model reproduced the variation of BMD observed in V667M patients, as shown by DXA measurements^(^ ^4^ ^)^ and is therefore relevant to estimate the effect of the V667M in humans. However, we could not evidence any modification of bone microarchitecture in mice, in contrast to what was observed in our patients or previously reported in *Lrp5* KO mice. Indeed, we cannot rule out a selection bias because patients were selected on low BMD and presence of fractures with no evidence of secondary osteoporosis. They could be considered as having a mild form of EOOP as the incidence of fracture was relatively low. Because the copy number variation or exome analyses were not performed, we cannot rule out that undetected variant or deletion in another gene could be responsible of low BMD in addition to the *LRP5* variant. The bone profile observed in patients could be related to other mutations in unexplored sequences such as introns, alterations in modifier genes, differences in epigenetic factors, or undescribed mutations in other genes. It is also possible that unknown nongenetic factors could play a role and explain why the phenotype was more severe than that observed in mice.

Altered bone microarchitecture was observed in *Lrp5* KO mice,^(^ ^12^, ^14^, ^15^, ^43^ ^)^ but the phenotype is expected to be stronger with a KO than with a single‐nucleotide mutation, as in this model. We cannot completely rule out an effect of the V667M variant on bone microarchitecture though, as modifications might be too small to be detected with our experimental plan, based on mice with a C57Bl/6 background, known to have one of the lowest bone mass among frequently used laboratory mice.^(^ ^44^ ^)^ In particular, the cortical thickness could be slightly reduced. As the low BMD and bone fragility could not be explained by impaired microarchitecture, we investigated the bone quality through estimation of its biomechanical properties. We observed that the stiffness and yield load appeared lower in *Lrp5* ^V667M^ mice. This could promote accumulation of microdamage in bone and therefore increase bone fragility. Lower stiffness is usually associated with a lower mineral content of the bone matrix.^(^ ^45^ ^)^ We could not evidence a clear reduction in the mineral‐to‐matrix ratio compared with CTL mice, but a slight reduction could exist as a trend was observed regardless of the method for organic matrix content estimation. A reduction in matrix mineralization could result from the reduced osteoblastic activity and be responsible for the low BMD observed. A more in‐depth analysis of the bone matrix showed a reduced hydroxyproline‐to‐proline ratio of collagen. A reduction in posttranslational proline hydroxylation was reported to favor overmodification of collagen chains and affect the stability of the collagen triple helix,^(^ ^46^ ^)^ the assembly of collagen fibrils,^(^ ^47^ ^)^ and could have an impact on mineralization of the bone matrix.^(^ ^48^, ^49^ ^)^ Mutations in *P3h1*, one of the enzymes responsible for hydroxylation of proline residues in collagen chains, are responsible for some forms of osteogenesis imperfecta.^(^ ^50^ ^)^ However, expression of *P3h1* that might alter the hydroxylation of collagen was not significantly different in our model. The exact mechanism through which the V667M variant of *LRP5* impacts proline hydroxylation warrants further investigation. Our findings provide new insights into mechanisms of bone fragility induced by mutations of *LRP5*. It highlights that *LRP5*‐related EOOP involves modifications in the quality of the extracellular matrix and further emphasizes the role of collagen posttranslational modifications. Interestingly, *LRP5*‐related EOOP has recently been classified in the same group as osteogenesis imperfecta in the classification of genetic skeletal disorders.^(^ ^22^, ^51^ ^)^ Our results strengthen the idea of a continuum between *LRP5* EOOP and osteogenesis imperfecta, as *LRP5* variants can be responsible for alterations of the collagen structure of bone extracellular matrix. The discrepancy between a marked osteoblastic phenotype and the mild bone phenotype was an unexpected finding and might involve compensation mechanisms and modification of signaling metabolic pathways.

Regarding the retinal vasculature, nonspecific vascular tortuosity was found in two patients, the significance of which remains uncertain at this point. Indeed, tortuosity can be observed in other diseases such as diabetes, high blood pressure, and obstructive sleep apnea.^(^ ^52^, ^53^, ^54^, ^55^ ^)^ Whereas high blood pressure was present in one of these patients, the other one did not have any history of these conditions. Also, arteriolar tortuosity was observed in the retinopathy of prematurity, the presentation of which is similar to FEVR,^(^ ^56^ ^)^ and arterial and venous tortuosity was also described in FEVR,^(^ ^57^ ^)^ which can result from *LRP5* mutations. Therefore, *LRP5* variants could have contributed to the observed tortuosity. The phenotype appeared clearer in mice with a higher rate of retinal vascular tortuosity in *Lrp5* ^V667M^ than in CTL mice. Vessel tortuosity can be related to disturbed blood flow, endothelial cell dysfunction, or hypoxia.^(^ ^53^ ^)^ As collagen type 1 is impacted in *Lrp5* ^V667M^ mice, it is conceivable that the vessel tortuosity in our murine model could be related to anomalies in collagen type IV, as in familial retinal arteriolar tortuosity, a condition linked to mutations in *COL4A1*.^(^ ^58^ ^)^ However in this condition, increased tortuosity of the second‐ and third‐order arteries is observed, whereas in the two *Lrp5* ^V667M^ patients, the tortuosity involved first‐order arteries.

In conclusion, the homozygous p.V667M low‐frequency variant of *LRP5*, and more generally loss‐of‐function mutations at heterozygous level of *LRP5*, can indeed have an impact on bone mineralization and alteration of the quality of the bone matrix. These results highlight the benefit of screening EOOP patients, investigating *LRP5* variant as a susceptibility factor of bone fragility. *LRP5* also plays a role in retinal vascularization development, and the V667M variant might increase retinal vascular tortuosity.

## Author Contributions

**Stéphanie Fabre:** Conceptualization; data curation; formal analysis; validation; writing – original draft; writing – review and editing. **Morgane Bourmaud:** Conceptualization; data curation; formal analysis; methodology; writing – original draft; writing – review and editing. **guillaume mabilleau:** Data curation; formal analysis; validation; writing – review and editing. **Ruben Goulet:** Data curation; formal analysis; writing – review and editing. **Aude Couturier:** Data curation; formal analysis; writing – original draft; writing – review and editing. **Alexandre Dentel:** Data curation; formal analysis; writing – original draft; writing – review and editing. **Serge Picaud:** Conceptualization; writing – review and editing. **Thomas Funck‐Brentano:** Data curation; formal analysis; writing – original draft; writing – review and editing. **Corinne Collet:** Conceptualization; formal analysis; writing – original draft; writing – review and editing. **Martine Cohen‐Solal:** Conceptualization; data curation; formal analysis; funding acquisition; methodology; project administration; resources; validation; writing – original draft; writing – review and editing.

## Conflicts of Interest

The authors report no conflicts of interest in relation to the work.

### Peer Review

The peer review history for this article is available at https://www.webofscience.com/api/gateway/wos/peer‐review/10.1002/jbm4.10741.

## Supporting information


**Appendix S1.** Supplementary Information

## Data Availability

The data that support the findings of this study are available from the corresponding author upon reasonable request.
