# Supplementary material for: Lrp5 p.Val667Met Variant Compromises Bone Mineral Density and Matrix Properties in Osteoporosis
Source: JBMR Plus. 2023 Mar 28;7(6):e10741. doi: 10.1002/jbm4.10741 (PMC10241090; doi:10.1002/jbm4.10741)
Supplement: Supplementary file 1 — Appendix S1. Supplementary Information [file JBM4-7-e10741-s001.docx]

**Supplemental materials**

Supplementary materials include supplemental methods, 4 figures and one table

**Supplemental methods:**

Analysis of bone composition in mice

Quantitative Backscattered Electron Imaging (qBEI) experiments were performed on right midshaft femurs embedded in methylmethacrylate. The blocks were carbon-coated and observed with a scanning electron microscope (EVO LS10, Carl Zeiss Ltd, Nanterre, France) equipped with a five-quadrant semiconductor backscattered electron detector. The microscope was operated at 20 keV with a probe current of 250 pA and a working distance of 15 mm. The backscattered signal was calibrated using pure carbon (Z = 6, mean gray level = 25), pure aluminum (Z = 13, mean gray level = 225), and pure silicon (Z = 14, mean gray
level = 253) standards (Micro-analysis Consultants Ltd, St Ives, UK). The cortical and trabecular bone areas were imaged at a 250X nominal magnification, corresponding
to a pixel size of 0.5μm/pixel. The entire cross-sectionnal cortical area was imaged by stitching multiple fields of view. Three variables were obtained from the bone mineral density distribution: Ca_mean_ as the average calcium concentration, Ca_peak_ as the peak calcium concentration, and Ca_width_ as the width of the histogram at half maximum of the peak.

For Raman microspectroscopic analysis, bone cross-sections of the femur midshaft used for qBEI were rapidly polished with a diamond paste to remove the carbon coating and affixed on a mirrored stainless steel slide (304L Stainless steel, Equinox 49, La Seguiniere, France). Three raman spectra were recorded between tetracycline and calcein labels with an inVia qontor confocal raman microscope with a holographic grating (1200 lines/mm) providing a spectral resolution of ~1cm^-1^ and a 785 nm laser source at 10 mW power (Renishaw, Marne-la-Vallée, France). Each spectrum was acquired as the average of three consecutive spectra with each collected for 20 seconds using a 50X objective (NA = 0.75), focusing the laser into a ~1.3 µm spot on each sample. The raman system was calibrated daily with an internal silicon sample to ensure wavenumber accuracy. Spectral pre-processing was performed with an in-house written script in Matlab R2021b (The mathworks, Natick, MA) that consisted in background fluorescence removal using a fourth-order polynomial fitting algorithm, pMMA contribution subtraction and smoothing with a Savitzky-Golay filter (degree 2, window size 9). The following physicochemical parameters were determined from spectra:

- Mineralization (mineral to matrix ratio) is the intensity ratio between the ν1PO_4_ (960 cm^−1^) peak and the matrix bands (amide I ~1667 cm^−1^, amide III ~1243 cm^−1^ or proline + hydroxyproline bands centred at ~854 cm^-1^ and ~872 cm^-1^, respectively).
- Mineral crystallinity was computed as the inverse of the full width at half maximum intensity of the ν1PO_4_ band (960 cm^−1^).
- Carbonate content was computed as the intensity ratio between the v1CO_3_ at ~1070 cm^-1^ and the v1PO_4_
- Collagen proline hydroxylation is the intensity ratio between the hydroxyproline band at ~872 cm^-1^ and the proline band at ~854 cm^-1^
- Nanoporosity as the area ratio between the pMMA contribution before subtraction (800-835 cm^-1^) and the amide III (1220-1315 cm^-1^)
- Relative proteoglycan (PG) content is the ratio of GAG/ CH3 (1365–1390 cm^−1^) to the amide III band.
- Relative pyridinoline content was calculated as the intensity at 1660 cm^-1^/area of the amide I (1620/1700 cm^-1^)

Evaluation of human retina

All patient underwent a detailed ocular exam including best-corrected visual acuity using a Snellen chart, slit-lamp biomicroscopy, measurement of intraocular pressure (IOP), and autorefractometry (TonoRef II; Nidek, Gamagori, Japan), and retinal imaging. All eyes underwent ultra-wide field color fundus photographs images obtained using Optos (Dunfermline, UK) and optical coherence tomography (OCT)-angiography (OCTA) using the Plex Elite OCTA device (Carl Zeiss Meditec Inc, USA). Ultra-wide field fluorescein angiography was also obtained in 6 of the 11 eyes using Optos (Dunfermline, UK).

OCTA images were obtained with the 100 kHz PlexElite 9000 Swept-Source OCTA device, which uses a swept laser source with a central wavelength ranged between 1040 and 1060 nm, a bandwidth of 100nm, an A-scan depth of 3 mm in tissue. The system operation speed is dependent on the scanning rate of the swept source (100 000 amplitude scan [A-scan] per second), and the axial and lateral resolutions in tissue are 6.3 μm and 20 μm, respectively. Image acquisitions were repeated if necessary to ensure images with good OCT signal penetration (signal strength >7) and minimal motion artifacts. Each 3x3 mm volume consisted of 300 B-scans of 300 A-scans repeated four times to generate OCTA images using an optical microangiography algorithm. Motion-related artifacts were minimized by an integrated line-scan ophthalmoscope eye tracker during data acquisition (FastTrac motion correction, Carl Zeiss Meditec, Inc, USA). *En face* OCTA images include superficial capillary plexus (SCP) and deep capillary complex (DCC) defined by the default automatic segmentation algorithm: the SCP slab was segmented between the inner and outer boundaries of the inner limiting membrane (ILM) and the inner plexiform layer (IPL), whereas for DCC the upper limit was at the level of IPL and the lower limit was defined by the outer plexiform layer (OPL).

To quantify the capillary density (CD) in the SCP and DCC, each 3x3mm *En face* OCTA image was converted into binary images using ImageJ (National Institutes of Health, Bethesda, Maryland, USA) according to previously reported methods (Kim AY 2016, Garrity ST 2017). First, images were processed with a top-hat filter (window size: 12 pixels). Then, each image was processed separately to create two distinct binarized images: one was processed with a Hessian filter, followed by global thresholding using Huang’s fuzzy thresholding method, and the other duplicate image was binarized through median local thresholding. Lastly, the two binarized images were combined, generating the final image in which only the pixels that existed on both binarized images were included. Using the binary image, CD was calculated as the ratio of the area occupied by vessels (white pixels) divided by the total image area (all pixels). In the SCP, we measured the CD after excluding the large retinal vessels (Campbell JP 2017).

- *Kim AY, Chu Z, Shahidzadeh A, Wang RK, Puliafito CA, Kashani AH. Quantifying Microvascular Density and Morphology in Diabetic Retinopathy Using Spectral-Domain Optical Coherence Tomography Angiography. Investig Opthalmology Vis Sci. 2016;57(9):OCT362.*
- *Garrity ST, Iafe NA, Phasukkijwatana N, Chen X, Sarraf D. Quantitative Analysis of Three Distinct Retinal Capillary Plexuses in Healthy Eyes Using Optical Coherence Tomography Angiography. Investig Opthalmology Vis Sci. 26 oct 2017;58(12):5548.*
- *Campbell JP, Zhang M, Hwang TS, Bailey ST, Wilson DJ, Jia Y, et al. Detailed Vascular Anatomy of the Human Retina by Projection-Resolved Optical Coherence Tomography Angiography. Sci Rep. mars 2017;7(1):42201.*

**Table 1:** **Description of clinical characteristics and genetic profile of** **EOOP patients with *LRP5* variants**

Clinical data were collected and BMD measured using a Lunar Prodigy device. Genetic analysis was performed using NGS panel, allele frequency was determined from the Exome Aggregation Consortium (ExAC) database, and probability of pathogenicity was assessed through the PolyPhen pathogenicity score.

F: female; M: male; HMZ: homozygous; HTZ: heterozygous.

| 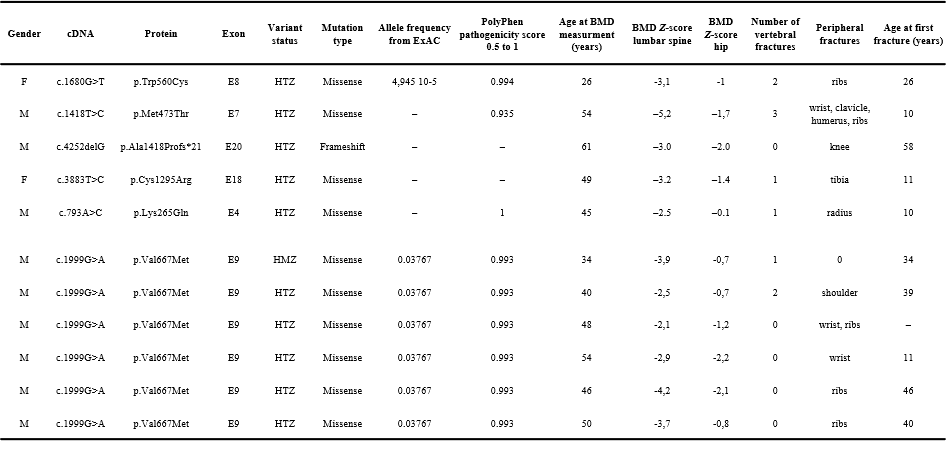 |
| --- |

**
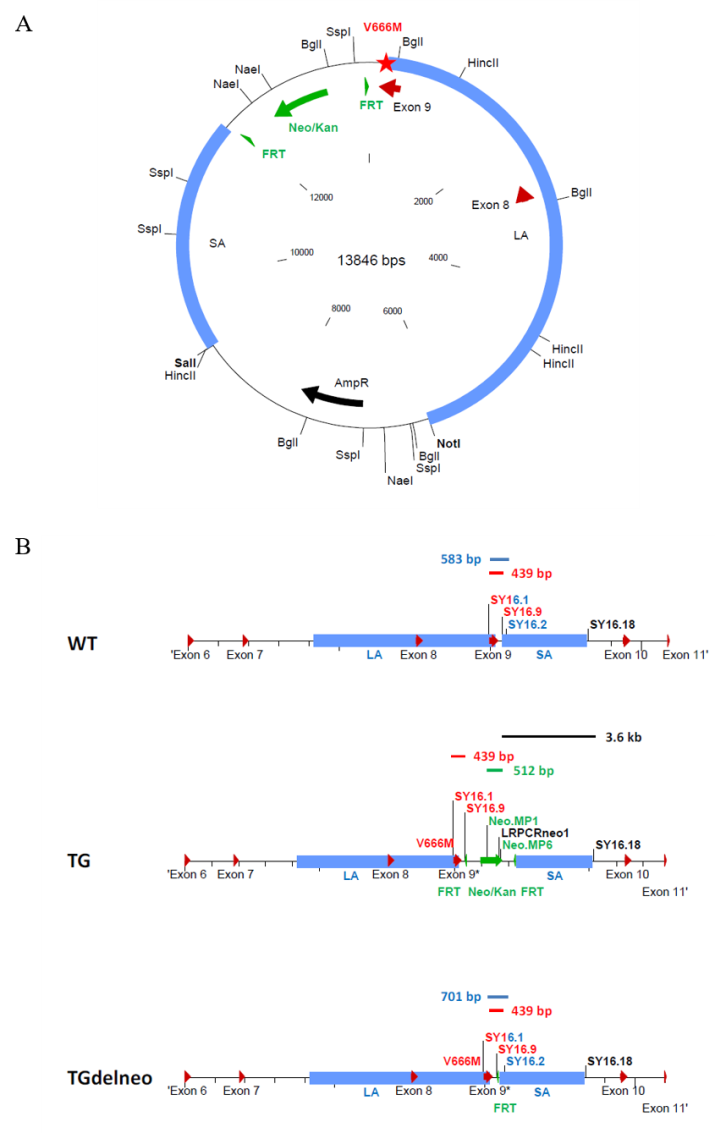
**

**Supplemental figure 1: Generation of the Lrp5^V667M^ murine model**

(A) Target vector for the V666M *Lrp5* mutation insertion. The base exchange (indicated by a red star) in exon 9 of Lrp5 (red arrows) is inserted together with the FRT-flanked neomycin resistance cassette (green). The long arm of homology has a length of 5.1 kb whereas the short arm of homology extends for 2.8 kb (LA and SA; blue boxes). Restriction enzymes used for confirmation are indicated. The targeting vector can be linearized with SalI or NotI prior to the electroporation.

(B) The different Lrp5 alleles: Lrp5 wild type (WT, upper panel), the targeted allele (TG, middle panel) and the targeted allele after deletion of the neomycin cassette (TGdelneo, lower panel). The binding sites of the primers used for genotyping are indicated in relation to the Lrp5 exons (red arrows) and the FRT flanked neomycin cassette (green). The corresponding PCR fragments are indicated in red (for mutation), green (for neo presence), and blue (for neo deletion) and black (for detection of homologous recombination). The V666M mutation inserted in exon 9 of Lrp5 (exon 9) is indicated in red.

**Supplemental figure 2:**

(A) Measurement of OS/BS: osteoid surface/bone surface in CTL and Lrp5^V667M^ mice. (B) Serum concentration of P1NP (N-Terminal propeptide of type 1 procollagen) and CTX (C-terminal telopeptide of type 1 collagen) in CTL and Lrp5^V667M^ mice.

**Supplemental figure 3:** mRNA relative expression of prolyl 3-hydroxylase 1 (*P3h1*), prolyl 3-hydroxylase 2 (*P3h2*), prolyl 4-hydroxylase subunit alpha-1 (*P4ha1*), prolyl 4-hydroxylase subunit alpha-2 (*P4ha2*) in tibia of control (CTL) and Lrp5^V667M^ mice.

**Supplemental figure 4:** **Retinal features in patients with Lrp5 variants**

(A) Representative ultra-wide field fundus images of retinal vascularization and tortuosity observed in 2 EOOP patients (arrow heads) compared to (B) normal individual. (C) Quantification of capillary density of the retina of control patients and EOOP patients carrying LRP5 variants.
